# Making programmes worth their salt: Assessing the context, fidelity and outcomes of implementation of the double fortified salt programme in Uttar Pradesh, India

**DOI:** 10.1111/mcn.13243

**Published:** 2021-07-18

**Authors:** Shruthi Cyriac, Amy Webb Girard, Usha Ramakrishnan, M. G. Venkatesh Mannar, Kriti Khurana, Rahul Rawat, Lynnette M. Neufeld, Reynaldo Martorell, Mduduzi N. N. Mbuya

**Affiliations:** ^1^ Doctoral Program in Nutrition and Health Sciences, Laney Graduate School Emory University Atlanta Georgia USA; ^2^ Global Alliance for Improved Nutrition New Delhi India; ^3^ Hubert Department of Global Health, Rollins School of Public Health Emory University Atlanta Georgia USA; ^4^ The India Nutrition Initiative New Delhi India; ^5^ Department of Chemical Engineering and Applied Chemistry University of Toronto Toronto Ontario Canada; ^6^ Bill and Melinda Gates Foundation Seattle Washington USA; ^7^ Global Alliance for Improved Nutrition Geneva Switzerland; ^8^ Global Alliance for Improved Nutrition Washington District of Columbia USA

**Keywords:** anaemia, coverage, double fortified salt, fidelity of implementation, iron deficiency, monitoring and evaluation, quality

## Abstract

Double fortified salt (DFS) has proven efficacy in addressing iron deficiency and anaemia, thus improving maternal and child nutrition outcomes. However, DFS delivery in large‐scale settings is less understood, with limited documentation of its fidelity of implementation (FOI). We assessed the FOI of the DFS intervention in Uttar Pradesh, India, to improve the design and implementation of such programmes that aim to reduce the anaemia burden, especially in women of reproductive age (WRA). We conducted in‐depth interviews with DFS programme staff (*n* = 25) and end‐user WRAs (23), guided by a programme impact pathway. We transcribed and thematically analysed the interviews and used an adapted analytic framework to document FOI across four domains—objects of intervention, implementation staff, implementation context and target of implementation. DFS utilisation remained low due to a combination of factors including poor product quality, distribution challenges, ineffective promotion and low awareness amongst end‐user WRAs. Motivation levels were higher amongst district‐level staff compared to frontline staff, who lacked supervisory support and effective incentives to promote DFS. Three typologies of DFS users emerged—‘believers’, ‘thrifters’ and ‘naysayers’—who indicated differing reasons for DFS purchase and its use or nonuse. The implementation of the DFS programme varied significantly from its theorised programme impact pathway. The adapted analytic framework helped document FOI and assess the programme's readiness for impact assessments and subsequent scale‐up. The programme needs product quality improvements, incentivised distribution and stronger promotion to effectively deliver and improve the realisation of its potential as an anaemia prevention strategy.

**Key messages**

- The double fortified salt (DFS) production, distribution and awareness creation process documented low fidelity, which influenced the perceptions of end‐users and ultimately their utilisation of the programme.
- The presence of ‘believers’ who used DFS, despite the organoleptic issues reported, suggests the potential for further expansion of coverage and utilisation.
- Several challenges identified in this programme can be resolved through improvements in product quality, including effective colour masking and encapsulation of the iron‐premix in DFS, and by improving motivation levels of the frontline programme staff.

## 1 INTRODUCTION

Anaemia is a widespread public health problem, affecting 1.93 billion people globally and commonly caused by iron deficiency (Kassebaum, 2016; Petry et al., 2016). It leads to debilitating effects that include adverse pregnancy outcomes (Allen, 2000) and impaired cognition in women and their offspring (Larson et al., 2017). The World Health Assembly (WHA) aims to address this by targeting a 50% reduction in the global anaemia prevalence amongst women of reproductive age (WRA) by the year 2025 (WHO, 2014). Several countries, in an effort to attain these WHA targets, have adopted staple food fortification—the addition of nutrients during commercial processing of foods such as cereals, salt and edible oil.

In India, the *‘Anemia mukt Bharat’* (Anaemia‐free India) campaign (n.d.) recommends fortification of staple foods with multiple nutrients, including iron, as an anaemia prevention strategy. Consequently, some states adopted and distributed double fortified salt (DFS)—salt fortified with iron and iodine—shown to reduce iron deficiency in controlled settings, through their existing social safety net programmes (SSNPs). Although distribution through SSNPs that reach vulnerable populations is a promising strategy to scale‐up fortification initiatives, its utility as a delivery platform remains to be evaluated and little is known about what influences DFS programme delivery in real‐world settings (Larson et al., 2021).

Addressing these evidence gaps requires documenting the fidelity of implementation (FOI) of DFS programmes, that is, whether programmes are implemented as intended. Conducting impact evaluations on programmes which have not been effectively implemented (Banerjee et al., 2018) can result in a Type III error (Dobson, 1980), which is a failure to identify if a lack of impact is due to the intervention itself or due to poor programme implementation (Carroll et al., 2007). Ensuring FOI in DFS programmes helps identify and resolve implementation challenges (Carroll et al., 2007) prior to conducting impact assessments, which is critical for programmes to realise their full potential. This increases their likelihood of translating initiatives to impacts (Ridde, 2016) and not abandoning the programme prematurely.

India implemented its flagship large‐scale DFS programme in Uttar Pradesh (UP) (Diosady et al., 2019) in 2017. Ten districts in UP, with a high anaemia prevalence, received DFS through the SSNP called Public Distribution System (PDS). Fair price shops (FPS) operating under the PDS were used as a DFS delivery platform. All FPS in the 10 districts distributed DFS at subsidised prices to nearly 3 million low‐income households (approximately 15 million individuals). A consortium led by the Global Alliance for Improved Nutrition, including St. John's Research Institute, Sanjay Gandhi Post Graduate Institute, Emory University, Cornell University and The India Nutrition Initiative, evaluated the programme. The team worked closely with the programme staff to gain experiential learning (Warren et al., 2020) for a process evaluation of the DFS programme, which included routine monitoring data collection and a midline evaluation. Using mixed‐methods data, the midline evaluation highlighted how the implementation unfolded (Mbuya et al., 2015; Rawat et al., 2013)—a quantitative survey revealed high DFS programme coverage but low utilisation (Cyriac et al., 2020). Based on this finding, we focused the qualitative research to examine experiences related to DFS use and programme delivery, unpacking some of the reasons for low utilisation in spite of high programme coverage.

In this paper, we present our analyses of the midline qualitative data, examining the UP DFS programme's FOI, and formulate recommendations that programme staff can use to adapt across intervention districts and inform the design and effective implementation of DFS programmes in other contexts, especially to improve the iron status of WRA.

## 2 METHODS

**Figure 1:**
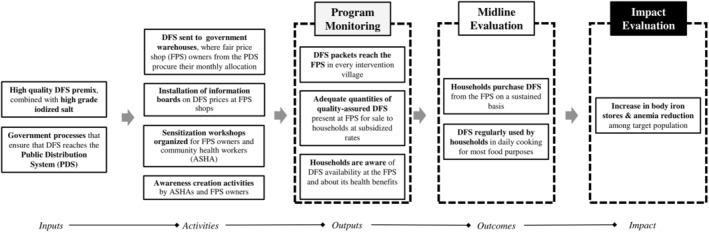
Theory based programme impact pathway: DFS programme in Uttar Pradesh, India. DFS, double fortified salt (with iron and iodine)

We developed a theorised UP DFS programme impact pathway (PIP) in collaboration with the DFS programme team (Figure 1). Developing this was an iterative process that involved several discussions with DFS implementation staff and review of programme documents, including the results framework and revisions to the logic model (Jadhav et al., 2019). The detailed pathway (supporting information Figure S1), developed through this process, articulates the intervention inputs and activities of the DFS programme (government processes, production and distribution of DFS, quality assurance and awareness creation) and their links to proximal and distal outcomes, and to final programme impact. The evaluation team used insights from the PIP to identify broad research areas for the midline evaluation. The routine monitoring process, led by the programme staff, provided insights about programme outputs, and the midline evaluation further explored these insights.

Briefly, the PIP (Figure 1) posited four components as key to successful programme implementation: high‐quality product, an efficient distribution mechanism, effective training of frontline programme staff and awareness creation amongst end‐users in households. We have a broad definition of ‘high quality’ of the DFS. It meets not only the exact chemical formulation of iron and iodine but also the colour masking and encapsulation requirements (Diosady et al (Diosady et al., 2019)) that make the ‘premix particles’ (iron premix added to iodised salt to produce DFS) similar to salt granules in appearance, even after cooking. Better awareness about DFS amongst end‐users could lead to sustained demand, subsequent purchase and continuous use of DFS, thereby ensuring impacts on nutritional and health outcomes.

### 2.1 Study sites and sampling

**Figure 2:**
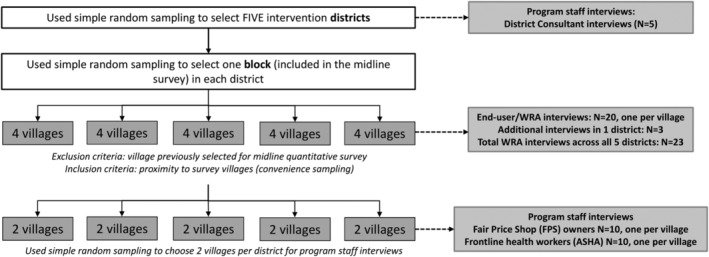
Midline qualitative interview sampling strategy. WRA, Women of Reproductive age; ASHA, Accredited Social Health Activist

We selected five programme districts for the midline evaluation (Figure 2), using a simple random sampling process. After interviewing the District Consultant (DC) (programme staff) in each district, we selected four villages per district for other interviews. We excluded villages that were part of the quantitative survey but used a convenience sampling approach to select those which were proximate to these excluded villages. In 10 out of the 20 villages, we interviewed a FPS owner and a community health worker (Accredited Social Health Activist [ASHA]). In villages where multiple FPS owners and ASHAs were present, one each were selected using a simple random sampling method.

Finally, we chose one household per village for the end‐user interviews, where the eligible respondent was a WRA (between 18 and 49 years), with a child between 6 and 59 months (same eligibility criteria as the midline survey). We considered code saturation (Hennink et al., 2017)—whereby no new insights emerge during data collection or when the themes identified in interviews begin to repeat—as the main principle for ascertaining sufficiency of the sample, and towards this end, three additional households were selected in one village in which we had not achieved saturation. We had a final sample of 48 interviews, across four types of respondents in five districts: FPS owners (*n* = 10), ASHAs (*n* = 10), end‐users (*n* = 23) and DCs (*n* = 5).

### 2.2 Data collection and analysis

Four research assistants received training on the overall DFS process evaluation, interview guides, qualitative interviewing and reflexivity. They were divided into two teams, consisting of an interviewer and a note‐taker. Semistructured interviews were then conducted in Hindi and audio‐recorded with participant permission. For interviews with FPS owners, ASHAs and DCs, the focus was on DFS programme‐related responsibilities and motivation levels. For the end‐user interviews, we examined DFS utilisation patterns and associated reasons for partial use or nonuse, probing on themes around meal preparation, salt usage and experience with DFS. Daily team debriefs were conducted to help address quality of data collection, refine the interview questions or probes, identify emerging themes and assess code saturation.

The in‐depth interview recordings were transcribed verbatim and translated from Hindi to English. We reviewed, de‐identified and uploaded the translated transcripts for analysis in MAXQDA. Using a thematic analysis approach, we first reviewed and memoed (Birks et al., 2008), that is, annotated, all interviews. A set of deductive codes were identified using the PIP with additional inductive codes developed using a data abstraction matrix implemented during daily team debriefs. Codes were further refined through additional review and memoing of transcripts. From this process, a preliminary coding framework was developed and applied to a sample of the transcripts. A second coder independently applied the same codebook to same interview transcripts, and the two coders discussed the process to adjust the framework and finalise the codebook. The first coder then applied the finalised coding framework to conduct thematic analysis.

### 2.3 Ethics

Institutional review boards at Sanjay Gandhi Post Graduate Institute of Medical Sciences, Uttar Pradesh, and Emory University, Atlanta, GA, reviewed and approved the data collection and analyses protocol.

### 2.4 Analytic framework

**Figure 3:**
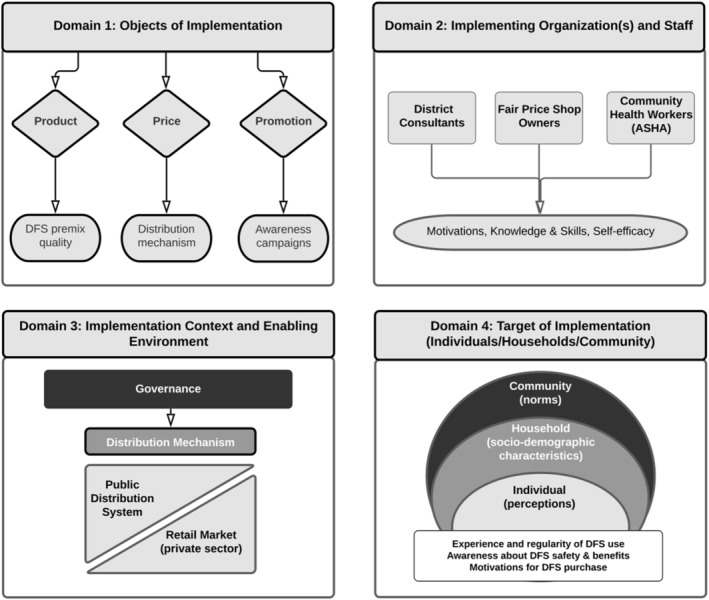
The adapted analytic framework of implementation science in nutrition. DFS, double fortified salt (with iron and iodine); ASHA, Accredited Social Health Activist

As a guide for our analysis, we relied on the Implementation Science in Nutrition (ISN) framework (Tumilowicz et al., 2019), adapted from the Consolidated Framework of Implementation Research (Damschroder et al., 2009), to fit diverse nutrition programme implementation contexts. After reviewing multiple implementation frameworks (Carroll et al., 2007; Durlak & DuPre, 2008; McIsaac et al., 2018; Menon et al., 2014; Meyers et al., 2012; Walugembe et al., 2019), both within and outside the field of nutrition, we selected the ISN framework because it is a more comprehensive framework that covers several elements beyond just the implementation process. While many frameworks touch upon one aspect of implementation, that is, assessing its fidelity (adherence, intervention delivery etc.), the ISN framework and our adaptation of it touches upon other important elements such as the organisational (motivations, knowledge and skills and self‐efficacy of key programme staff) and policy (enabling environment) contexts of implementation in addition to examining the implementation process. We adapted the ISN framework as an analytic tool to examine the implementation process and document FOI in the UP DFS programme across four domains (Figure 3). Specifically, under Domain 1, we examined three objects of implementation: (1) *Product*, assessed by the quality of the premix (including colour masking and its encapsulation); (2) *Price*, examined through the procurement and distribution of the intervention through the PDS; and (3) *Promotion*, operationalised through awareness creation strategies. Under Domain 2, we used interview data with DCs, FPS owners and ASHAs to examine how their motivations, knowledge and skills, and levels of self‐efficacy influenced intervention delivery, thereby affecting the FOI. For Domain 3, we used emerging themes from interviews with DCs and FPS owners to understand the implementation environment that affected DFS distribution. Finally, Domain 4 focused on the target of implementation, that is, end‐users who were individuals (nested in households and communities), and how they perceived the programme. Specifically, we examined how community norms, household socio‐demographic characteristics and individual perceptions influenced end‐user interactions with the DFS programme. Here, Domain 1 (objects of implementation) affected end‐user experience, Domain 2 (programme staff) influenced their DFS awareness levels and Domain 3 (programme context) affected their motivations for DFS purchase.

## 3 RESULTS

We present the findings from our thematic analyses and include some illustrative quotes in‐text. Additional quotes are in Table 1, organised by thematic domains, themes and subthemes. In Table 2B, we present a comparative analysis of DFS perceptions by different typologies of DFS end‐users.

### 3.1 DOMAIN 1: Objects of implementation (product, price and promotion)

#### 3.1.1 Product

*DFS product quality was compromised due to inadequacies in premix production*. The DFS production technique, developed at University of Toronto, was transferred to India for scale‐up in the UP programme (Diosady et al., 2019). In theory, the iron compound was to be sized to match salt granules, colour‐masked and encapsulated to ensure that it remains inconspicuous in salt. The local salt manufacturer was then supposed to procure the premix, mix it with iodised salt and distribute the DFS to households in the programme districts through the PDS (Figure 1). In reality, processes related to premix procurement and DFS production by salt manufacturers were largely opaque, and enforceable controls on premix quality standards were absent (Moorthy & Rowe, 2021). Even though the product met stipulated quality specifications as per the Indian standards, these pertain only to the chemical content of the premix and do not provide regulations on colour masking or encapsulation. Consequently, inadequacies in colour masking and encapsulation of the premix particles were common, making it easily distinguishable from salt granules.

*“Something is mixed which looks black in colour. When you open it [the DFS packet] you will see the black granules which when dissolved makes food black”—* as quoted by a DFS end‐user

#### 3.1.2 Price

*FPS owners implemented alternative distribution strategies to recover costs incurred in DFS procurement and transport*. The PDS distributed subsidised rations—rice, wheat and kerosene fuel—every month to low‐income households through the FPS network. In the 10 DFS programme districts, the UP government added the DFS as an additional item to the PDS. Every household with four or less members received one DFS packet (1 kg) and those with more than four members received two. The government procured and transported PDS rations and DFS to a decentralised network of warehouses. Each programme district had warehouses present in different blocks (smaller administrative area), and FPS owners purchased their monthly ration and DFS quota from their respective block‐level warehouses. DFS quantity allocation for every FPS was predetermined by the government, and FPS owners were required to deposit the full payment in advance.

With several households finding “*black*” premix particles in DFS, FPS owners found it challenging to sell the product and recover costs. While procurement and transportation costs incurred for high‐demand items like grains and kerosene were easily recovered, DFS sales were neither incentivised nor were expenses reimbursed (Table 1, Subtheme: quantity and costs). Moreover, DFS purchase orders were never adjusted for lower demand, forcing FPS owners to procure all allotted quantities irrespective of stockpiles already held in the shop. Consequently, FPS owners adopted strategic cost‐recovery measures such as bundling, where DFS and other highly desired rations were sold as a package instead of individual items (Table 1, Subtheme: DFS bundling).

*“They will have to take the salt…they at least have to take one packet of salt….those who don't take 2 packets, they take one. [But if they refuse to take even one] then I won't give ration.”—* as quoted by a FPS owner

#### 3.1.3 Promotion

*In spite of a programmatic push for DFS promotion, trainings for FPS owners and ASHAs had limited effectiveness*. An overreliance on awareness creation was necessitated with end‐users finding conspicuous premix particles in DFS. However, the programme was unprepared to incentivise promotion activities or invest in a dedicated workforce to support the more intensive DFS promotion efforts (Table 1, Subtheme: intensity). Therefore, in the absence of effective communication, end‐users finding “black” premix particles and related food discolouration viewed it as a deterrent from continuing DFS use.

FPS owners were provided an information board, with details about DFS prices and available quantities for households, and a one‐time block‐level training was conducted. During these training sessions, FPS owners were asked to proactively inform DFS end‐users to anticipate a darkening of foods and to share strategies such as altering the timing of salt addition while cooking to alleviate the discolouration. Trainers used examples of food darkening while cooking in cast iron pans in an effort to normalise it, and reinforced messages around safety and benefits of DFS. However, some FPS owners found training sessions to be less credible as officials from the PDS department did not routinely attend. Additionally, follow‐up of actions promised at the training went unfulfilled, causing some FPS owners to believe that the session was held only as a “*formality*”.

*“Some officers had come at the block‐level and had conducted a training session…they were telling us that we have to enforce this thing [DFS], and we have to [create] awareness [among] the people about this salt. In that training, they had given us a lunch packet, a diary, and a pen. After that, all formalities were completed. They had said, ‘We will come to your village, we will do meeting and street‐plays through which we will make people aware about DFS’. It has been seven to eight months since they said that, but no one has come till now”—* as quoted by a FPS owner

ASHAs also attended a training session at the inception of the programme, where they were informed about the DFS programme and its benefits. However, not all ASHAs recalled attending a DFS‐specific training and confused it with their routine job trainings from the health department that often included topics related to iodised salt. DFS‐specific message recall was poor amongst these ASHAs (Table 1, Subtheme: ASHA training), and follow‐up trainings with DCs did not take place after the programme inception. We found that the DFS training sessions lacked specificity around message content and target households. ASHAs were simply asked to add DFS communication onto their other message creation efforts during home visits but not provided any job aids or incentives for taking on this additional work.

### 3.2 DOMAIN 2: Implementing organisation and staff

#### 3.2.1 District‐level staff

*DCs had strong faith in the DFS programme and believed that the programme needed more time to become successful*. They were highly motivated individuals who had complete confidence in the programme, maybe naively so.

*“This is a nice program and it should continue…It is really a good program as it is concerned with eradicating anaemia. If this will continue in a proper manner, then the problem of anemia will definitely be solved”—* as quoted by a District Consultant

They had a vision for programmatic success, believing that accepting a new product like DFS will take time in the community (Table 1, Subtheme: DC vision). They were accountable for the overall implementation, with responsibilities across two levels. At the block‐level, DCs visited warehouses to monitor DFS supply and collect DFS samples for laboratory assessments of levels of iron and iodine. They also liaised with FPS owners to resolve any supply disruptions or procurement lags and organised the one‐time training session for FPS owners and ASHAs at the time of inception (Figure 1). At the community level, DCs visited FPS owners to collect routine monitoring data on DFS sales and discuss any implementation challenges faced at the local level. DCs also conducted short exit interviews at the FPS, with end‐users, to collect programme monitoring data on awareness and use of DFS.

#### 3.2.2 Frontline staff—Motivation

*DFS trainings failed to motivate some FPS owners*. In spite of attending DFS training sessions and being knowledgeable about the programme benefits, most FPS owners wanted to reduce their responsibilities, with some believing that ASHAs should play a bigger role as DFS is intended as a health intervention.

*“If the main focus of this program is to eradicate anemia, then DFS should have been distributed by community health centers and ASHAs. The only work of a FPS owner is to distribute the rations to the public. Who uses DFS and who doesn't ‐ we have no business with that.”—* as quoted by a FPS owner

They were demoralised and frustrated about being forced to both procure and sell DFS. They wanted a reduction in DFS quantities by 50% and introduction of other subsidised commodities, such as soap or detergent, in its place. FPS owners found that most end‐users raised problems with food discolouration due to the “*black*” premix particles, even after telling them about DFS benefits. They felt that the product quality needed improvements, and addressing discolouration issues will make it easier for them to sell DFS.

#### 3.2.3 Frontline staff—Beliefs and self‐efficacy

*Awareness creation efforts by FPS owners and ASHAs were influenced by their knowledge and perceptions about the programme, and levels of self‐efficacy*. The FPS owners we interviewed had varying degrees of success in DFS promotion. A few of them adopted DFS promotion strategies such as highlighting the use of DFS in their own homes, thereby building community's trust in the product. They leveraged their training knowledge to effectively communicate strategies to minimise discolouration and address safety concerns, thus encouraging many people to utilise DFS (Table 1, Subtheme: successful awareness creation). Other FPS owners, who did believe in the DFS benefits but held a more paternalistic view, tried to convey the message that the government intervention is meant to improve the health and well‐being of the people. However, this group of FPS owners failed to recognise the agency of end‐users to accept or reject a new product and therefore could not effectively connect with their communities (Table 1, Subtheme: unsuccessful awareness creation). The same training messages on DFS benefits, safety and strategies to minimise discolouration had a much lesser impact in this context. A few FPS owners lacked the self‐efficacy to push messages around DFS benefits to the public without support and advocated for a stronger awareness campaign in villages led by others (Table 1, Subtheme: low confidence).

Most ASHAs appeared to have greater self‐efficacy and were aware of the premix in DFS. Several ASHAs communicated DFS benefits to community members during and after their house visits for routine immunisation and prenatal checkups. They were confident communicators and were able to address most people's apprehensions about DFS, convince them about the need to consume DFS to prevent anaemia and goitre.

*“Sometimes the people also question us, “why do you tell us these things?”, but we explain everything to them after which they understand. Among 10–12 people there are 1 or 2 who don't want to understand our words, but when they see that the people near them are using DFS they also start to use it. There are some people [with whom] if we explain a little, they understand it, but there are others [for whom] it is very hard to explain about DFS. When we explain to them repeatedly, they understand everything…that DFS contains iron and iodine…but there are some rich people who are not ready to eat DFS…For them, it does not matter that we explain or not…Some people are totally rigid that DFS is not good, so they don't eat it. They are not ready to accept DFS no matter what. They eat the iodized salt which is available in the [retail] market. Otherwise, all other people eat DFS”—* as quoted by an ASHA

Three ASHAs we interviewed were unaware about the iron content in DFS, but nonetheless noted promotion of DFS use in their communities in an effort to address goitre.

### 3.3 DOMAIN 3: Implementation context and enabling environment

The overall implementation context and policy environment had three implications for the UP DFS programme. First, the policy context made it challenging to address some of the implementation issues. The current regulatory standards focus on the chemical composition of DFS and need to be broadened to include colour masking, encapsulation and other production aspects that affect consumer acceptance of the product. Enforcing product quality controls will remain difficult from a programmatic perspective, unless the regulatory standards and their enforcement are strengthened to include not just the safety but also the physical appearance of DFS. Second, the DCs who liaised with FPS owners to resolve supply and distribution challenges found it difficult to address their concerns, due to the administrative nature of the PDS. There were long feedback loops with multiple officials in the government and bureaucratic channels often delayed incentivising FPS owners or addressing their concerns (Table 1, Subtheme: bureaucratic process). Third, some FPS owners believed that cardholders viewed the PDS as a SSNP providing subsidised products that were of lower quality and considered their full priced retail market alternatives as more aspirational.

*“People think that the government is giving a sub‐standard thing…it is given at the FPS for free or INR 2 or 3, and they think that it is rubbish”* – as quoted by a FPS owner

*“If DFS is also sold at INR 20 in the market, then the people will riot to buy it from me…it has come directly to me, people don't understand the value. If it is sold in the market, then the public will think it is a very good salt”* – as quoted by a FPS owner

**Table 1:** Thematic analysis of domains 1–3, using the adapted analytic framework of implementation science in nutrition

Theme	Subtheme	Quote
**Domain 1—objects of implementation**		
**Price**	Quantity and costs	**FPS owner:** *No one wants to take DFS on his own will…since we are getting the allotment, we have to helplessly tell them to take it. From that salt we don't get any commission…sometimes we find torn salt packets, and we have to bear the amount of those because no one takes torn packets. In a family even if one person is there, he is also given with one kg of salt in a month…so in total, he/she is given 12 kg of salt in a year…so how will one person use those 12 packets of salt in a year?”*
	DFS bundling	**FPS owner:** *There was a meeting by the FPS owners to tell [higher authorities] that we won't take DFS. But then he [the official] said that it is compulsory to take DFS, we were bound to take that. They say if DFS is not taken, then don't give them the ration. They tell us to distribute it [DFS] anyhow…distribute it to even to those people who do not have a [PDS] card. First we try to convince them [PDS cardholders], but if they do not agree we have to put in a condition that we will not give the ration*.
**Promotion**	Intensity	**DC:** *There is only one weapon of IEC [information, education & communication]*
		**DC:** *ASHAs do this [DFS promotion], but they have a heavy work load…they are working for DFS, but can't focus much*. D*uring ration distribution, they [FPS owners] communicate with those who have some complaints. [But] because they have to collect the money, maintain the PDS card, fill out forms…they already have a high work load…if we expect that he will make each and every one understand [about DFS benefits and safety], then it can't be possible…we can't force them too, because they already have lots of work*.
	ASHA training	**ASHA:** *We were informed that it is an iodized salt and the people should eat this. In the training they said this salt is very good, it increases the stamina of ours, so we must eat it. This is a salt with small particles and it contains iodine, calcium*, etc. *I was given the responsibility that we have to inform by doing door to door campaign. We tell households that they should eat this salt because it contains black elements and provides strength to the body, “If you will eat this salt then it will give more benefits to your health, it will give strength to your body”. Most of the people might be knowing about this. They might be eating it…but we don't know because we only tell them [about DFS] but don't go inspect whether they eat it or not.”*
**Domain 2—implementing organisation and staff**		
**Motivation**	DC vision	**DC:** *For improving the implementation of this DFS program more time and manpower is needed…and the second thing is incentives for FPS owners…incentives also for community health workers. Look, ASHA is an incentive‐based worker and without any incentive she will not do anything*.
**Knowledge and skills**	Successful awareness creation	**FPS owner:** *I told them that the black content is iron and that it will address anaemia in our bodies. Some agreed, some did not. At the start, even my family members said the curry becomes black. I asked them to cook in iron utensils using any other salt, ‘You will know why it becomes black. Cook in iron utensils using any salt and keep it for some time’. So we removed people's apprehensions by saying that you make food in iron utensils and keep it there for some time. It will also become black. Then the people got to know that it [food darkening] is because of iron. Almost 50% households are using [DFS]…those who see that we are eating [DFS] and that we are not having any side‐effects*.
	Unsuccessful awareness creation	**FPS owner:** *We also tell people to put this salt after the dal or curry is done…so that the color of the curry or dal does not turn too black, but they don't obey our instruction…so what can we do with this?*
		**FPS owner:** *It is only black in color, who looks in the evening? Eat it. What is the problem?*
**Self‐efficacy**	Low confidence	**FPS owner:** *There should be awareness campaigns for the public…doctors from government hospitals should be present and teams should be stationed in villages to spread the information. We, FPS owners, will support it completely. The benefits of DFS should be explained and the general public should be informed. If we, FPS owners, tell about its benefits nobody will trust us. If others put in [DFS promotion] efforts, then it will be successful. Since I own and operate my shop, I will always tout my products as good…the public will think that since DFS is coming to us, we have to sell it somehow…they will think that I am trying to sell or get rid of DFS*.
**Domain 3—implementation context and enabling environment**		
**Governance**	Bureaucratic process	**DC:** *We assure the FPS owners that discussions are going on in the administration and it [change] will come as soon as it is implemented*
	PDS vs. Retail	**FPS owner:** *If DFS is also sold at INR 20 in the market, then the people will riot to buy it from me… it has come directly to me, people don't understand the value. If it is sold in the market, then the public will think it is a very good salt*

Therefore, they suggested a simultaneous introduction of DFS through privatised retail markets, hoping that this signal be lucrative to FPS owners for their own DFS promotion.

### 3.4 DOMAIN 4: Target of implementation (individuals, households, community)

Community perceptions about the government influenced individual engagement with the DFS programme. Some households revered and trusted the government, leading them to purchase and use DFS. Others remained fearful or frustrated, causing them to seek out alternate sources of information and validation regarding DFS safety after noticing the “*black*” premix particles.

We identified three emerging typologies of the end‐users for the DFS programme, based on their perceptions about the programme, awareness about DFS benefits, experience with DFS and subsequent engagement with the intervention. We classified these typologies as ‘believers’ (*n* = 4), ‘thrifters’ (*n* = 10) or ‘naysayers’ (*n* = 9). They had similar socio‐demographic characteristics (Table 2A), and we compare their responses including representative quotes about DFS perceptions in Table 2B.

**Table 2A:** Descriptive characteristics (*n*) of DFS end‐users, by typology

Characteristics	‘Believers’ (*n* = 4)	‘Thrifters’ (*n* = 10)	‘Naysayers’ (*n* = 9)
***District‐wise distribution(n)***			
*Auraiya (4)*	1	3	‐
*Etawah (7)*	1	4	2
*Faizabad (4)*	1	1	2
*Mau (4)*	‐	‐	4
*Moradabad (4)*	1	2	1
***Average age***	25.9	26	25.2
***Education level***			
*No schooling*	‐	2	1
*Primary school*	2	3	4
*Secondary school*	‐	3	‐
*High school*	‐	‐	2
*Graduate level*	2	2	2
***Average number of children***	2.8	2.8	1.9
***Average household size***	7	7.3	7.1

**Table 2B:** Thematic analyses of domain 4 ‐ target of implementation (individuals/households/communities)

Theme (subtheme)	Typologies of DFS users		
	‘Believers’ (*n* = 4)	‘Thrifters’ (*n* = 10)	‘Naysayers’ (*n* = 9)
**Perception about DFS** (Trust in the program)	**Believer Quote (BQ) 1:** *Everyone in our neighborhood is eating that salt. We think that the government is doing it for our benefit. We just don't know what is in it*.	**Thrifter Quote (TQ) 1:** *We have to use it since we are getting it. We hope that it is for our benefit. And that is why we eat it*.	**Naysayer Quote (NQ) 1:** *If something like the Tata salt [retail brand] comes, then everyone will eat it. We don't know what is being mixed in DFS*.
			**NQ 2:** *Only money matters to them. They [FPS owners] bring DFS for money only*.
**Motivation for DFS purchase** (Bundling of DFS with PDS rations)	**BQ 2:** *We are eating it just because the government is providing it. [We are using] DFS and we haven't used crystal salt since then. I don't like DFS…[we still consume] maybe because it is available at a cheaper rate. We cannot just throw it because we are purchasing it with our money*.	**TQ 2:** *They give it [DFS] to us forcefully, they tell that if you don't take this salt then we don't give you kerosene also. So, how can we refuse for the salt? Kerosene is essential for our fuel. They [FPS owners] give this [kerosene] to the rich people only, but they force the poor people to take the salt. They are telling that these packets are coming for you…so we have to give these packets to you*.	**NQ 3:** *They give us forcefully. If they'll give us forcefully, then what can we do? They say if you'll not take salt, then we'll not provide you ration*.
**Awareness about DFS** (DFS contents and its benefits)	**BQ 3:** *My mother in law says that DFS is beneficial to health*	**TQ 3:** *It has iron. Maybe because of the tiny black crystals which is there. Because of it only the vegetables become black. They [public] don't understand this and many houses throw it. It is written over here [in the DFS packet]…if you read it you will know*.	**NQ 4:** *When I used it, it settled down and only the powder portion came up. So once I put it in a glass of water, it floated on the top and some red colored element settled down in the bottom. By eating that, people have stone in their stomach*.
	**BQ 4:** *There are least chances of getting diseases from its use. It is a good salt…pure salt…The doctor said that it is good for health and digestion*.	**TQ 4:** *It feels as if there is ‘Nirma’ [local detergent] in the salt…it looks blue black*.	**NQ 5:** *It gets black because something is mixed in it. I don't know what it is but DFS has vitamins and iodine. These all things are mixed in it but because of the taste and colour no one eats it. It is written on the packet that iodine is mixed in it, it is good for health and the body. But then no one likes to eat it in the family….when you open it you will see the black granules which when dissolved makes it black*.
**Experience of DFS:** (Organoleptic issue – Color)	**BQ 5:** *It [DFS] has something of black colour in it that darkens the dishes. It is just that the colour of the curry turns black…slight dark in colour*	**TQ 5:** *It destroys the food…the food color becomes black due to DFS. So people do not eat this…If it remains normal then everyone will eat this salt*.	**NQ 6:** *It makes the vegetable black…completely [black]…If we make DFS fine by grinding with jeera [cumin seeds] and ajwain [carom seeds], then also it becomes black. In any pulses or anything when you put it then the colour of the turmeric vanishes and only black is left. So it is not all good to see…At home they say, “the vegetable is not good, it is black what have you done?!”*
		**TQ 6:** *When any relative comes over, it is an embarrassment to serve a black vegetable. It won't look good, and it doesn't even feel good. When you make potatoes then even the cauldron even becomes black*.	
**Experience of DFS:** (Organoleptic issue – Taste)	**BQ 6:** *The taste is not that bad. We have been using it for so long, nothing has happened to us yet, and its taste is overall good*.	**TQ 7:** *When we add this salt in our food, our spices become watery instead of thick. It seems like something fell in it. When we add this salt in our curry we don't get the taste of any ingredients, meaning we don't get the taste of vegetables or spices ‐ everything became tasteless*.	**NQ 7:** *When you see that the vegetable is black…so you don't like it and it settles in the mind that the vegetable is not good in taste…When you put it [DFS] in vegetables, it tastes like water ‐ bland. The vegetables start melting, [as in] it gets overcooked, and if you just serve it in the plate then water releases because of the use of this salt. The iodized [Tata] salt doesn't make the vegetable bland, we use that salt from the very start so we are accustomed to its taste*.
	**BQ 7:** *This salt tastes something when the dish is freshly prepared and tastes something else after the dish has cooled down*	
**Experience of DFS:** (Perceived side effects)	**BQ 8:** *It's been around a year since DFS is being given…we have been using it since then. Other people complain that it caused rashes and such. But me and my kids are using it since then and don't have any complaints about it*	**TQ 8:** *It causes itching. This salt doesn't suit me. When I eat this salt, I start itching. Everyone doesn't get itching. Just that it suits someone and doesn't suit someone else. My brothers‐in‐law and my father‐in‐law eat it. Nothing happens to them. It doesn't suit only me. May be I got [rashes] due to [an] allergy. He [the doctor] said it's due to allergy and to protect myself from sun and water…I eat DFS, but in a very low quantity. I use crystal salt [in dishes] and if more is needed then a bit of that salt [DFS]*.	**NQ 8:** *Everyone had that itchiness; even our little child had rashes*.
			**NQ 9:** *When they gave that salt to us, we used it but when it started causing rashes, we stopped using it. The whole village suffered from rashes. Nobody is using that. If it suits them, they eat and if it doesn't, they don't eat. When we used that salt, rashes appeared and when we stopped using it, then rashes also disappeared*.
**Regularity of DFS use** (Usage in food)	**BQ 9:** *There are two ways of using salt. For wet food I use crystal salt after cleaning it. For dry food I use the government's [DFS]*.	**TQ 9:** *We don't like DFS but sometimes we eat it when crystal salt gets finished. Because DFS contains very small particles, we can't wash it. If we will wash it then it will get dissolved with the water. In some packets of salt we find some particles which look like stone*.	**NQ 10:** *That salt [DFS] makes us unhealthy…We don't like this. About 2 to 3 months ago, we were eating DFS, but now we are not eating it*.
	**BQ 10:** *We use DFS when the salt is less in food [to sprinkle on top]*.		
**Regularity of DFS use** (usage other than food)	**BQ 11:** *We eat DFS, we also add it to buffalo feed and still have some DFS remaining*	**TQ 10:** *We wouldn't throw it. We use it sometimes in the fodder that we feed the buffaloes*.	**NQ 11:** *If they [other villagers] have buffaloes, then they feed it to their buffaloes. But we don't have any cattle; we don't have agricultural land, so we don't have any cattle…We just throw it away in the pond…6‐8 packets*.
		**TQ 11:** *Because this salt is turning the food black, people throw this salt in agricultural fields…Once seven packets of salt were left over in our home for a long time, so we also threw those DFS packets in the field like other people. We put it in the field because the paddy is affected by insects*.	
**Mitigation strategies** (Overcoming organoleptic experiences)	**BQ 12:** *It blackens the food when added. It blackens the yellow colour of dal. But still we use it…for yellow colour we add more turmeric so that it hides the blackness of salt*	**TQ 12:** *We have to use the filter to get the pure salt. We put it in water, the black thing settles down and becomes like lime…tight…it is not able to mix fast….and we use the salt that remains*.	**NQ 12:** *Dal becomes less black and the vegetables becomes more blackish in colour. It is visible in the curry with spices. If we consider palak [spinach] curry, it does not become black because it is already dark in colour. But in curry with spices which are yellow in colour, they become black. The program should take such a step so that the food will look good…it shouldn't look black. There is no problem in the taste*.

#### 3.4.1 Believers

‘Believers’ considered DFS as beneficial to health and adopted mitigation strategies to overcome any adverse organoleptic experiences. ‘Believers’ were mostly convinced that the DFS programme was introduced by the government for their benefit *(*Table 2B *, Believer Quote [BQ]: 1)*.

“Everyone in our neighborhood is eating that salt. We think that the government is doing it for our benefit …” — BQ1

They considered DFS to be a “*good salt*” *(BQ 4)* and had a positive attitude towards DFS after hearing about its benefits from either the local doctor/ASHA or the FPS owner in their village. While DFS bundling was a reason some of them purchased DFS *(BQ 2)*, all ‘believers’ used DFS in most, if not all, their food preparation. During cooking, they found that DFS caused food to turn *“slight[ly] dark in color*” *(BQ 5)* but continued its use because of its benefits *(BQ 3)*. Some ‘believer’ households adopted strategies to mitigate discolouration, for example “*add more turmeric*” *(BQ 12)* or sprinkling DFS only on top of prepared food instead of adding it while cooking. One ‘believer’ found that the taste altered *“after the dish has cooled down” (BQ 7)*, but none of the believers perceived any side‐effects from DFS use *(BQ 8)*.

#### 3.4.2 Thrifters

‘Thrifters’ purchased DFS only because it was bundled with other commodities and used DFS when they could not afford to purchase other salt in the retail market *(*Table 2B *, Thrifter Quote [TQ] 2)*.

“We have to use it since we are getting it. We hope that it is for our benefit …” – TQ2

While some knew of DFS' iron content *(TQ 3)*, most had no awareness about the “*tiny black crystals*” and considered these to be limestone particles or “*Nirma*” (local detergent brand), based on its texture *(TQ 4)*. ‘Thrifters’ predominantly preferred and used *kada namak* (crystal salt), a cheap locally available unrefined salt, for all their food and beverage requirements. Crystal salt had to be washed and crushed before use; some families mixed in DFS with the crushed salt, while others simply stored DFS as a back‐up option to be used when they ran out of readily crushed crystal salt *(TQ 9)*. A few of them used “*the filter to get the pure salt*” from DFS packets by separating the premix particles *(TQ 12)* and some others kneaded dough with DFS, to make *rotis (*flatbread*)* or *pooris* (deep‐fried flatbread), where discolouration was minimal. They avoided using DFS while cooking vegetables or lentils as the discolouration was more prominent, especially when the food was not consumed immediately after cooking. They hesitated serving discoloured food to guests and relatives, saying “*it doesn't even feel good*” *(TQ 6)*, and family members were ashamed to open their packed *tiffin* (lunch) in front of others due to the dark colour. One user also noted that DFS watered down the food and *“everything became tasteless” (TQ 7)*. Some family members in ‘thrifter’ households experienced “*itching*” after DFS use *(TQ 8)* and therefore refrained from DFS use. However, many recognised that these rashes may not be caused by DFS use but could be due to external factors (exposure to sun or seasonal allergy).

#### 3.4.3 Naysayers

‘Naysayers’ had apprehensions about DFS and mostly used “*Tata salt*” which was available in the retail market *(*Table 2B *, Naysayer Quote (NQ): 1)*.

*“If something like the Tata salt [retail brand] comes, then everyone will eat it. We don't know what is being mixed in DFS.” –* NQ1

After initially trying DFS, ‘naysayers’ found the food discolouration unacceptable, saying “*the program should take such a step so that the food will look good*” *(NQ 12)*. Most of them had no awareness about DFS contents *(NQ 4, NQ 5)* and considered it to be mixed with impurities, such as “*pebbles*”. A few ‘naysayers’ also found that cooking with DFS made their food bland as it led to “*melting*” of vegetables *(NQ 7)*. Some thought that DFS made their food bitter, but one participant mentioned that this could be a perception that “*settles in the mind*” after seeing the food discolouration *(NQ 7)*. Another participant mentioned that DFS makes her family “*unhealthy*” *(NQ 10)* and two ‘naysayer’ households reported that everyone in their family, *“even our little child”*, suffered from rashes *(NQ 8, NQ 9)*. Unlike ‘thrifter’ households, ‘naysayers’ believed that it was DFS use that caused rashes and completely discontinued its use. In spite of non‐use, these participants had to continue purchasing DFS due to PDS bundling *(NQ 3)*. As DFS stock accumulated in their homes, they mixed DFS in cattle feed *(NQ 11)* and/or scattered it in agricultural fields to address worm infestations.

## 4 DISCUSSION

Uttar Pradesh is one of the most densely populated and impoverished Indian states which, despite several efforts, has made little or no progress in its nutritional status in the last decade. The level of anaemia in the state is 53% in nonpregnant WRA and 63% in preschool aged children (International Institute of Population Sciences, 2015) and over 70% of them have iron deficiency anaemia (Ministry of Health and Family Welfare, UNICEF, & PopulationCouncil, 2019). To address this issue, the UP government introduced the DFS intervention in 10 districts across the state, which were purposively chosen by the government based on a high anaemia prevalence.

Prior to conducting an impact assessment of this DFS programme, we considered it essential to first establish the FOI (Carroll et al., 2007; Durlak & DuPre, 2008; Habicht et al., 1999; Kim et al., 2015; Robert et al., 2006) by proactively assessing percieved product quality and measuring programme coverage. Our documentation of the FOI in the UP DFS programme revealed important differences between what was intended as per design in the PIP and what was actually implemented in the programme districts. Using the PIP, the FOI in the UP DFS programme was monitored across (1) product quality, (2) the distribution mechanism, (3) awareness creation and (4) end‐user perspectives. Applying the ISN framework as an analytic guide, we explored why there was low utilisation of DFS (Cyriac et al., 2020) and observed three emerging typologies of DFS end‐users who had varied experiences with the programme.

DFS quality compromises affected the overall FOI and resulted in low utilisation levels, with only ‘believers’ continuing to use DFS in most or all foods and adopting mitigation strategies to overcome discolouration issues. The scale‐up of DFS interventions in India was emboldened by the successful implementation of universal salt iodisation (Diosady et al., 2019). The latter's accomplishment was mainly due to it being a passive intervention, requiring minimal behaviour change to switch to using iodised salt. While the DFS formulation used in the UP programme was designed to remain equally passive in theory, DFS that was eventually produced at scale, procured and distributed through the PDS had conspicuous premix particles. Cooking with DFS led to food discolouration, therefore making it a less desirable product for end‐users of the UP programme.

Bundling of DFS was a cost‐recovery strategy adopted by FPS owners to cope with low product demand, where the sale of all other subsidised rations was made conditional upon DFS purchase. This bundling, coupled with food discolouration issues, led DFS end‐users to perceive it as a poor product. With several households purchasing DFS only to obtain the other subsidised PDS rations, purchase rates stayed high but failed to translate to equally high utilisation. This highlights a missed opportunity, because the DFS programme did manage to effectively reach the most vulnerable end‐users but did not convert ‘thrifters’ and ‘naysayers’ into regular DFS users. Some FPS owners suggested alternate distribution of DFS through retail markets, perhaps due to the difficulties faced by them to recover DFS costs. Although similar market‐based strategies have shown to be successful in other contexts (Banerjee et al., 2017), an expansion of DFS distribution though private markets might be premature unless there are improvements in the product quality.

Training of frontline staff was originally designed to be a one‐time activity, requiring low time and resource investments. This would have been sufficient had the premix maintained high quality. However, DCs had to quickly adapt their training sessions to better suit implementation realities. FPS owners and ASHAs were trained to proactively address the DFS‐led food discolouration and encourage behaviour change to minimise discolouration, in addition to reiterating the safety and benefits of using DFS. However, our results suggest that there were differences in attitudes, perceptions, motivations and self‐efficacy levels in FPS owners and ASHAs, which argue for a segmented training approach with these two groups of frontline workers to ensure that the needs and motivations of each are addressed.

DFS promotion efforts had limited success in creating awareness around product benefits/safety and increasing product demand/desirability. Best practices in nutrition behaviour change highlight how successful interventions integrate strong promotion campaigns (Jacob Arriola et al., 2020). These interventions demonstrate the need to conduct multiple home visits (Olney et al., 2015), or incentivise ASHAs (Avula et al., 2013; Sarma et al., 2020; Suchdev et al., 2010), organise interactive cooking demonstrations (Robert et al., 2006) and tasting sessions (Loechl et al., 2009) or provide supportive supervision (Kim et al., 2015; Sarma et al., 2020) and refresher training sessions. DFS promotion strategies did not originally plan or budget to do this, and consequently, these efforts did not increase utilisation levels. It is important to acknowledge here that while DFS promotion efforts are important to normalise food discolouration experienced in UP, they can only be a short‐term solution in improving implementation outcomes. The sustainable solution would be to improve product quality, and the DFS production technology is continuously evolving (Baxter & Zlotkin, 2015; Diosady et al., 2019; Hurrell, 2021) to attain this, perhaps future programmes will not face the discolouration challenges observed in UP.

Although we demonstrate the use of the ISN framework as an analytic tool in this paper, using it as a design framework to rigorously assess programme implementation and/or design more effective delivery may have additional utility. It is important to also highlight that the sampling of interview participants for this qualitative research was restricted to rural areas, and several stakeholders linked to the programme may not have been interviewed.

## 5 CONCLUSION

The UP DFS programme faced implementation challenges that were identified and addressed, to the extent possible, during routine programme monitoring and the midline evaluation. However, some bottlenecks remained unresolved and continued to influence the FOI of the programme; product quality improvements were essential, frontline staff needed incentives and streamlined training and promotion efforts (albeit a stop gap measure) required higher investments in the interim. With the UP DFS programme documenting a low FOI, subsequent impact assessments were conducted only in a subsample of intervention districts where the potential for impact was higher, based on DFS utilisation estimates from the midline quantitative survey, carefully desiged to maintain objectivity and evaluation rigour. As new fortification programmes are implemented in multiple contexts, our approach of using the ISN framework to document programme fidelity can be replicated or modified to evaluate implementation outcomes and support rigorous programme design to acheive sustainable impact. We hope that this use of implementation research to course correct programmes can maximise their potential in addressing the anaemia burden in women and children, and ensure that DFS programmes are worth their salt.

## CONFLICT OF INTEREST

Authors have no conflicts to declare. This study was funded by the Bill & Melinda Gates Foundation and the project was implemented by The India Nutrition Initiative (TINI). Co‐authors employed by the Bill & Melinda Gates Foundation and TINI provided feedback on drafts of this manuscript. However, TINI and the funders of the study had no role in study design, data collection, data analysis, or the decision to submit the paper for publication.

## FUNDING INFORMATION

Bill and Melinda Gates Foundation.

## CONTRIBUTIONS

SC, KK, LMN and MNNM designed the study; SC, KK and MNNM were involved in the acquisition of the data; SC, AWG, RM and MNNM contributed to the analysis and interpretation of the data; SC wrote the paper, all authors provided critical feedback on the draft, all authors approved the final version submitted for publication.

## Supporting information


**Figure S1.** The Uttar Pradesh DFS Program Impact Pathway

## Data Availability

The data that support the findings of this study are available from the corresponding author upon reasonable request.
