# Supplementary figures and images for: Making programmes worth their salt: Assessing the context, fidelity and outcomes of implementation of the double fortified salt programme in Uttar Pradesh, India
Source: Matern Child Nutr. 2021 Jul 18;18(1):e13243. doi: 10.1111/mcn.13243 (PMC8710122; doi:10.1111/mcn.13243)

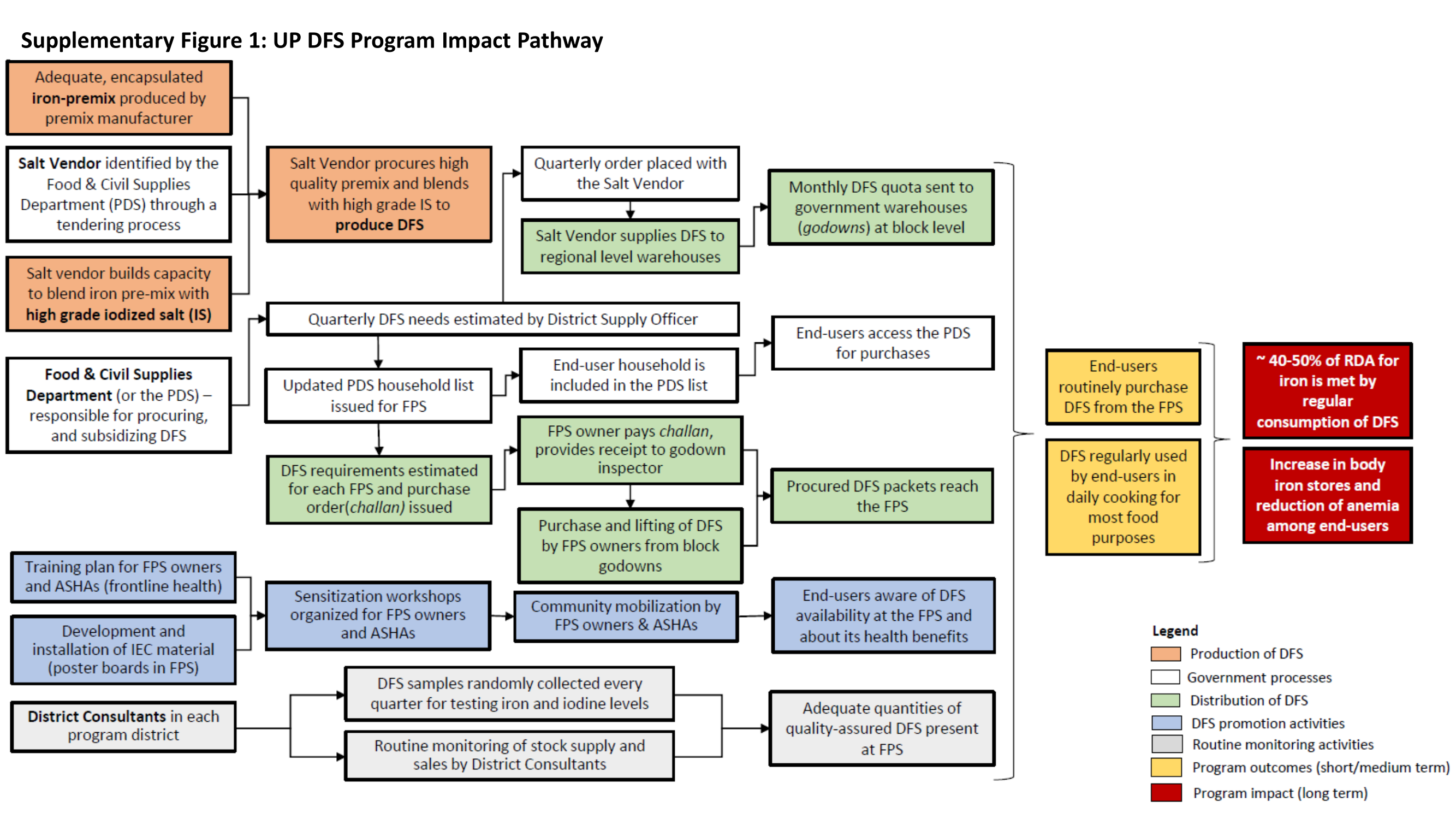

Supplement: Supplementary file 1 — Figure S1. The Uttar Pradesh DFS Program Impact Pathway [file MCN-18-e13243-s001.tiff]
